# Development and Validation of a Nomogram and a Comprehensive Prognostic Analysis of an LncRNA-Associated Competitive Endogenous RNA Network Based on Immune-Related Genes for Locally Advanced Rectal Cancer With Neoadjuvant Therapy

**DOI:** 10.3389/fonc.2021.697948

**Published:** 2021-07-19

**Authors:** Fang-Ze Wei, Shi-Wen Mei, Zhi-Jie Wang, Jia-Nan Chen, Hai-Yu Shen, Fu-Qiang Zhao, Juan- Li, Ti-Xian Xiao, Qian Liu

**Affiliations:** Department of Colorectal Surgery, National Cancer Center/National Clinical Research Center for Cancer/Cancer Hospital, Chinese Academy of Medical Sciences and Peking Union Medical College, Beijing, China

**Keywords:** locally advanced rectal cancer (LARC), neoadjuvant therapy, nomogram, competitive endogenous RNA (ceRNA) network, prognostic model

## Abstract

Colorectal cancer (CRC) is a common digestive tract tumor worldwide. In recent years, neoadjuvant chemoradiotherapy (CRT) has been the most comprehensive treatment for locally advanced rectal cancer (LARC). In this study, we explored immune infiltration in rectal cancer (RC) and identified immune-related differentially expressed genes (IRDEGs). Then, we identified response markers in datasets in GEO databases by principal component analysis (PCA). We also utilized three GEO datasets to identify the up- and downregulated response-related genes simultaneously and then identified genes shared between the PCA markers and three GEO datasets. Based on the hub IRDEGs, we identified target mRNAs and constructed a ceRNA network. Based on the ceRNA network, we explored prognostic biomarkers to develop a prognostic model for RC through Cox regression. We utilized the specimen to validate the expression of the two biomarkers. We also utilized LASSO regression to screen hub IRDEGs and built a nomogram to predict the response of LARC patients to CRT. All of the results show that the nomogram and prognostic model offer good prognostic value and that the ceRNA network can effectively highlight the regulatory relationship. hsa-mir-107 and WDFY3-AS2 may be prognostic biomarkers for RC.

## Introduction

Colorectal cancer (CRC) is the most commonly diagnosed digestive tract cancer in the world. Rectal cancer (RC) accounts for one-third of newly diagnosed CRC and is associated with poor prognosis (1). Many studies have recommended neoadjuvant chemoradiotherapy (CRT) as the standard treatment for locally advanced rectal cancer (LARC) because of its low toxicity and low metastasis rate (1). However, only approximately 15–27% of patients achieve a pathological complete response (pCR) (2), and most stage II/III RC patients receive surgery or adjuvant therapy. Due to the different responses to CRT, management of the response to CRT is significant for LARC patients (3). Therefore, it is necessary to construct a prediction model or study the mechanism of the response of LARC to CRT.

According to some studies, the TME is related to the amounts of *Fusobacterium nucleatum* (4) and interleukin (IL)-17A (5). Many studies have indicated that the TME plays an important role in the development and clinical outcomes of CRC (6–8). The TME also plays an important role in the clinical outcomes of LARC. There is some evidence showing that the presence of PD-1 and tumor-infiltrating lymphocytes before therapy can result in a better prognosis (9), and the high phospho-Drpl level in the TME of RAGE-G82S polymorphism patients is linked to the treatment effect (10). The components of the tumor microenvironment (TME) include stromal cells, immune cells, cytokines and other kinds of cells. Numerous studies have shown that TME cells play important roles in tumor progression, therapeutic effects and clinical outcomes and interact with each other (11–13). Therefore, it is important to explore the differentially expressed genes that link with the TME and respond to CRT in LARC patients. In our study, we explored the TME score and tumor-infiltrating immune cells (TICs) in RC and identified the differentially expressed genes (DEGs) between normal and tumor tissues (14). Based on the DEGs, we explored immune-related genes (IRGs) in RC. We utilized principal component analysis (PCA) to identify the key markers of IRGs in the GSE68204 dataset (15). Moreover, we utilized robust rank aggregation (RRA) to identify genes related to the response to CRT that were up- and downregulated simultaneously in three datasets from the Gene Expression Omnibus (GEO) database (16, 17). Then, we explored the hub genes that were shared with the key markers by PCA and RRA.

There is much evidence showing that competing endogenous RNAs (ceRNAs) play important roles in human cancers (18). The ceRNA network can clearly express the intricate interplay among mRNA, miRNA and lncRNA, which provides a perspective to explore the mechanisms of genes (19, 20). In our study, we identified target mRNAs based on the hub IRDEGs and built a ceRNA network to explore the regulation of lncRNA–miRNA–mRNA interactions in LARC after CRT. Based on the ceRNA network, we found two prognostic biomarkers for RC and utilized Cox regression to build a prediction model. Based on the hub IRDEGs, we also utilized least absolute shrinkage and selection operator (LASSO) regression to further screen the genes (21, 22) related to the response to CRT and constructed a predictive nomogram (23).

## Materials and Methods

### Gene Expression Datasets

The microarray datasets used in our study were downloaded from the TCGA and GEO databases. The RNA data of TCGA-READ (**Supplementary Files 1**–**3**), which contained two control tissues and 84 RC tissues with clinical data, were downloaded from the TCGA database (http://cancergenome/.nih.gov/). Three GEO datasets of LARC patients who received CRT, GSE93375 (24), GSE119409, and GSE139255 (**Supplementary Files 4**–**6**) were downloaded from the GEO database (http://www.ncbi.nlm.nih.gov/geo/).

### TME Score and Immune Infiltration

We utilized the R packages “estimate” and “limma” to estimate the proportions of stromal and immune cells in RC and calculated the ImmuneScore, StromalScore, and ESTIMATEScore of tumor tissues (25). Then, we divided the three datasets into high- and low-score groups and utilized Kaplan–Meier analysis to plot the survival curves through the R packages “survminer” and “survival”. We also utilized CIBERSORT to explore the relative percentages of immune cell types in RC (26). We utilized gene microarray immunofluorescence double staining, which was performed on rectal cancer diagnosed in Cancer Hospital in China, to validate immune infiltration. The CD4 rabbit mAb (48274S, CST, Danvers, MA, United States) was used as the antibody for CD4^+^ T cells, and the CD68 XP rabbit mAb (76437S, CST) was used as the antibody for M0 macrophages. The process of immunofluorescence double staining is shown in the **Supplemental Materials and Methods**.

### Hub IRDEGs

We utilized the R packages “edgeR” (27) and “limma” to separate mRNAs and lncRNAs and then explored the DEGs between normal and tumor tissues of RC in TCGA database according to the criteria |log(fold change)|>2 and P-value <0.01. We downloaded IRGs from the IMMPORT database (https://www.immport.org/) and identified IRDEGs using the R package “limma” (28). We downloaded the GSE68204 matrix files from the GEO database (**Supplementary File 7**) and divided the samples into three groups: normal, tumor-responsive, and tumor-non-responsive tissues. Based on the IRDEGs, we utilized PCA to explore the principal IRDEGs in GEO68204 for LARC patients who received CRT. Samples from the GSE119409 and GSE139255 datasets were classified according to clinical information; GSE93375, TRG1, and TRG2 were considered responders, and TRG3, TRG4, and TRG5 were considered non-responders (Mandard’s classification). Then, we identified differentially expressed genes between responder and non-responder groups of three matrix files in the GEO datasets utilizing the R package “limma”. We explored the genes that were upregulated and downregulated simultaneously (P < 0.05 and |log(fold change)|>0) using the RRA method with the R package “RobustRankAggreg”. We explored the hub IRDEGs of LARC using a Venn diagram through the online tool “VENN” (http://bioinformatics.psb.ugent.be/webtools/Venn/). The hub IRDEGs were the intersecting genes screened by PCA and RRA (29). We also utilized the online tool SangerBox and the R package “psych” to explore the relationships between the hub IRDEGs.

### GO and KEGG Enrichment Analyses

We utilized the R packages “org.Hs.eg.db”, “enrichplot”, “ggplot2”, “enrichplot”, and “GOplot” to conduct GO and KEGG pathway enrichment analyses, which satisfied the criterion of an adjusted P-value <0.05 to explore the biological values of principal IRDEGs screened by PCA and hub IRDEGs.

### Construction of the ceRNA Network and Prognostic Model

The miRNA and lncRNA matrix files of RC were downloaded from the TCGA database, and we identified the differentially expressed miRNAs (DEmiRNAs) and differentially expressed lncRNAs (DElncRNAs) with the R package “edgeR”. Based on the hub IRDEGs, we identified candidate mRNAs as targets of DEmiRNAs that were recognized by TargetScan, miRTarBase, and miRDB simultaneously (29). Then, we explored the interactions between DElncRNAs and DEmiRNAs. Based on the interactive relationships of DEmiRNAs–DElncRNAs and DEmiRNAs–DEmRNAs (30), we constructed a lncRNA–miRNA–mRNA ceRNA network and utilized Cytoscape software 3.6.1 to visualize the network. Based on the DElncRNAs and DEmiRNAs, we explored survival-related biomarkers with the R packages “survival” and “q value” in the TCGA-READ database. We utilized Cox regression analysis to build the prognostic model through the R package “survival” based on the survival-related biomarkers and utilized ROC curves and K–M plots to verify the prediction model. In addition, we utilized immune cells in the tumor infiltration database downloaded from TIMER (https://cistrome.shinyapps.io/timer/) to explore the correlations between the expression of immune cells and the risk score of the model.

### Validation of Differential Expression of Prognostic Biomarkers

We utilized qPCR to validate the differential expression between tumor and control tissues performed on eight pairs of RC and matched normal tissues. Total RNA was extracted from tissue samples. The RNA levels were calculated using the ΔΔCT method. The specimens were collected in Cancer Hospital in China, and this study was approved by the ethics committee at Cancer Hospital. All individuals in this study provided informed consent. The details of the process are provided in the **Supplemental Materials and Methods**.

### Development and Validation of the Prediction Nomogram

Based on the hub immune response-related DEGs, we utilized LASSO regression to screen the response-related factors in GSE68204 with the R packages “survival” and “glmnet”. Based on the factors identified, we built a nomogram through the R package “rms” to build a response-related prediction nomogram and used decision curve analysis (DCA), calibration curves, receiver operating characteristic (ROC) curves, and concordance index (C-index) to validate the nomogram (31, 32) through the R packages “Hmisc”, “ROCR”, “rms”, and “rmda”. To further validate the nomogram, we utilized bootstrapping validation (1,000 bootstraps) to calculate the verification C-index (23).

## Results

### TME of RC

**Figure 1:**
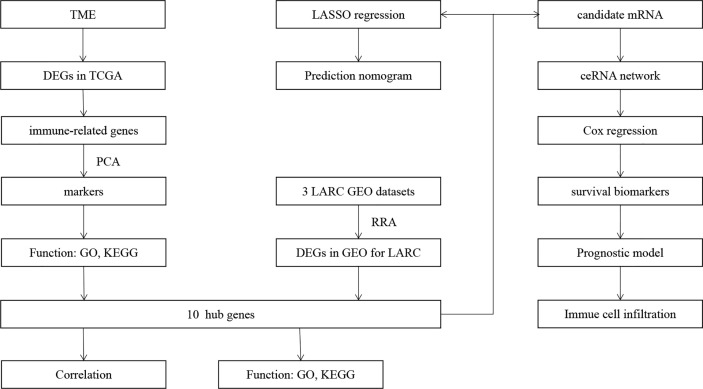
Flowchart of this study.

**Figure 2:**
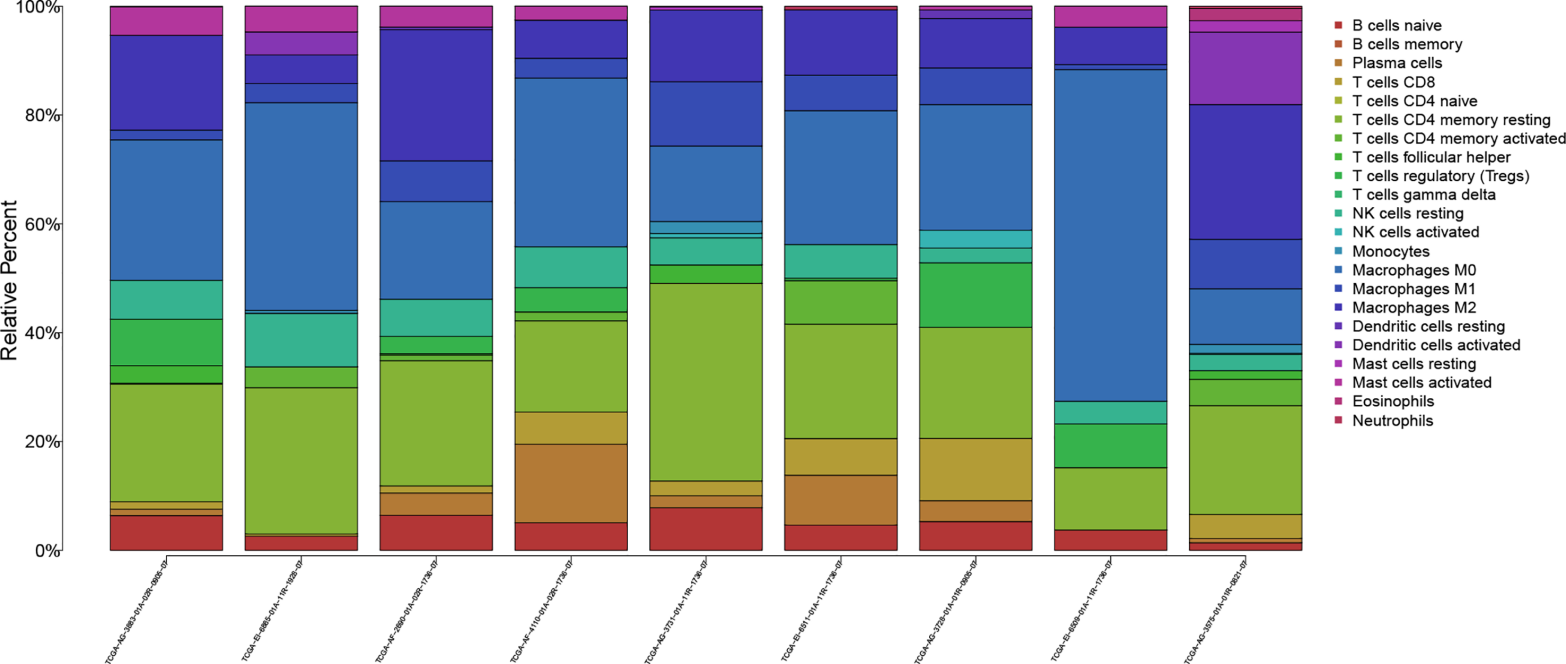
The proportions of 22 kinds of TICs in rectal tumor samples.

**Figure 3:**
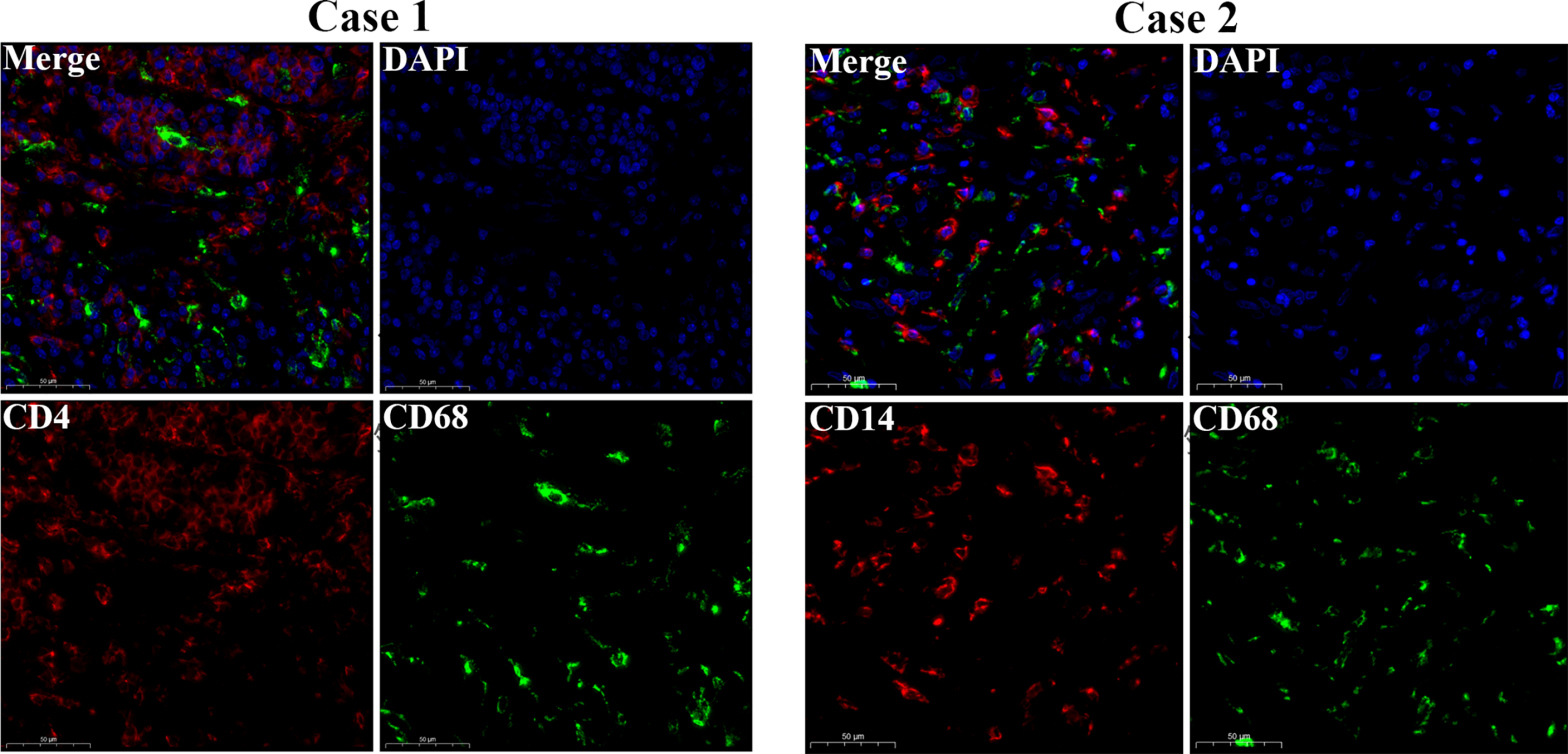
Gene microarray immunofluorescence double staining. Double immunofluorescence staining for CD14 (red) and CD68 (green) antigens of tumor cell. Scale bars = 50 µm.

The workflow of our study is shown in **Figure 1**. We analyzed three TME scores, the ESTIMATEScore, ImmuneScore, and StromalScore, and explored their relationships with clinical characteristics. There were no significant differences across different stages and TNM grades. We divided the three scores into two groups, and the K–M plot showed no significant survival benefit (**Supplementary Figures S1**–**S4**). As shown in **Figure 2**, CD4^+^ T cells and macrophages accounted for the highest relative percentages in RC tissues. The expression of CD4^+^ T cells and also macrophages also enriched on gene microarray of RC is shown in **Figure 3**.

### Identification of the Hub Immune-Related DEGs in LARC

**Figure 4:**
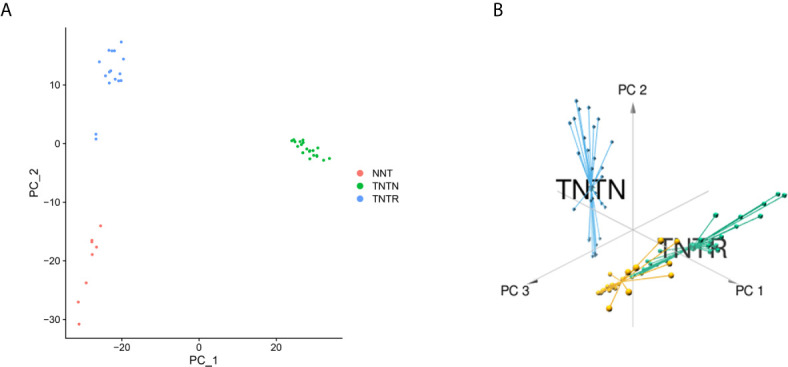
Identification of principal CRT-related genes through PCA. **(A)** PCA could separate immune-related genes in GSE68204. Orange dots represent the normal (NNT) group; green dots represent the tumor-non-responsive (TNTN) group; blue dots represent the tumor-reponsive (TNTR) group. **(B)** 3D distribution plot of PCA. Orange lines represent the normal (NNT) group; green dots represent the tumor-responsive (TNTR) group; blue dots represent the tumor-non-responsive (TNTN) group.

**Figure 5:**
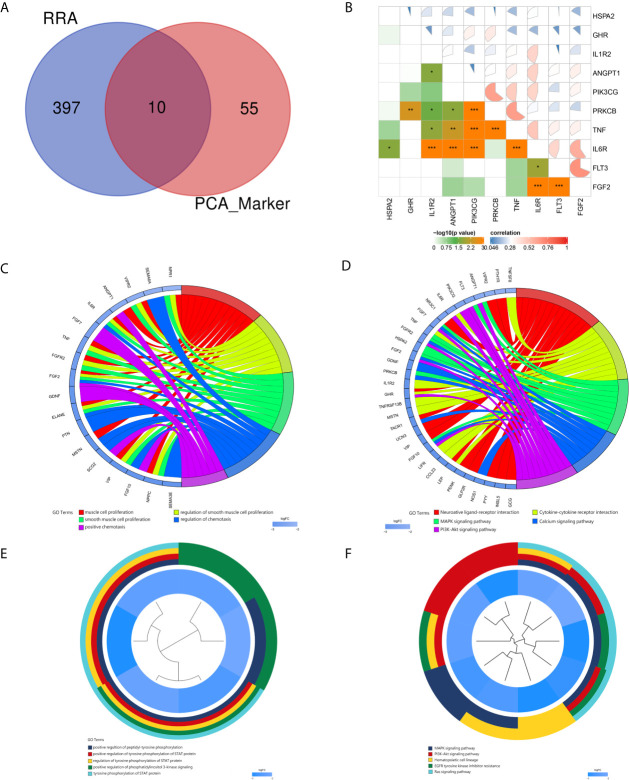
Identification and correlation of hub immune-related DEGs. GO and KEGG analyses of the markers and hub immune-related DEGs. **(A)** Venn diagram of hub immune-related DEGs. Blue indicates RRA of the three datasets obtained from the GEO database. Red indicates markers identified by PCA. **(B)** Correlations of hub immune-related DEGs. **(C)** Circ plot indicating the top five GO enrichment terms of markers. **(D)** Circ plot indicating the top five KEGG pathway terms of markers. **(E)** Cluster plot showing the top five GO enrichment terms of hub immune-related DEGs. **(F)** Cluster plot showing the top five KEGG pathway terms of hub immune-related DEGs.

Based on the RC mRNA matrix files downloaded from the TCGA-READ dataset, we first identified DEGs between control tissues and tumor tissues. A total of 107 IRDEGs were explored based on the DEGs. Then, we divided GSE68204 into three groups: normal, tumor-responsive, and tumor-non-responsive tissues. Sixty-five principal IRDEGs were identified based on the IRDEGs through PCA (**Figures 4A, B** **)**. As shown in **Figure 5A**, there were 10 hub IRDEGs (TNF, HSPA2, PRKCB, PIK3CG, GHR, IL1R2, FLT3, FGF2, IL6R, and ANGPT1) that were shared between PCA and RRA. The correlation heatmap showed that the hub IRDEGs had strong relationships with each other (**Figure 5B**). The characteristics of the datasets are shown in **Table 1**.

**Table 1:** Baseline information of datasets from the GEO database.

Dataset	No. of Responders to CRT	No. of Non-responders to CRT	No. of Normal tissues	Platform ID	No. of Row Perl Platforms
GSE68204	27	32	21	GPL6480	19,582
GSE93375	8	14		GPL15207	20,072
GSE119409	15	41		GPL570	21641
GSE139255	89	67		GPL22330	784

GSE, Gene Expression Omnibus Series; GPL, Gene Expression Omnibus Platform.

### GO and KEGG Enrichment Analyses of DEGs

For the markers recognized by PCA, the top five GO enrichment terms were muscle cell proliferation (P = 6.54E-10), regulation of smooth muscle cell proliferation (P = 7.66E-09), smooth muscle cell proliferation (P = 8.47E-09), regulation of chemotaxis (P = 8.66E-08), and positive chemotaxis (P = 1.12E-07) (**Figure 5C**). The top five KEGG enrichment terms were neuroactive ligand–receptor interaction (P = 8.92E-08), cytokine–cytokine receptor interaction (P = 9.88E-06), MAPK signaling pathway (P = 6.50E-05), calcium signaling pathway (P = 9.63E-05) and PI3K–Akt signaling pathway (0.000267) (**Figure 5D**).

For the hub immune response-related genes, the top five GO enrichment terms were positive regulation of peptidyl-tyrosine phosphorylation (P = 2.71E-08), positive regulation of tyrosine phosphorylation of STAT protein (P = 3.80E-08), regulation of tyrosine phosphorylation of STAT protein (P = 8.28E-08), positive regulation of phosphatidylinositol 3-kinase signaling (P = 9.08E-08), and tyrosine phosphorylation of STAT protein (P = 9.51E-08) as shown in **Figure 5E**. The top five KEGG enrichment terms were MAPK signaling pathway (P = 4.01E-07), PI3K–Akt signaling pathway (P = 1.20E-06), hematopoietic cell lineage (P = 4.15E-06), EGFR tyrosine kinase inhibitor resistance (P = 0.00010), and Ras signaling pathway (P = 0.00012) (**Figure 5F**).

### The ceRNA Network of LARC

**Figure 6:**
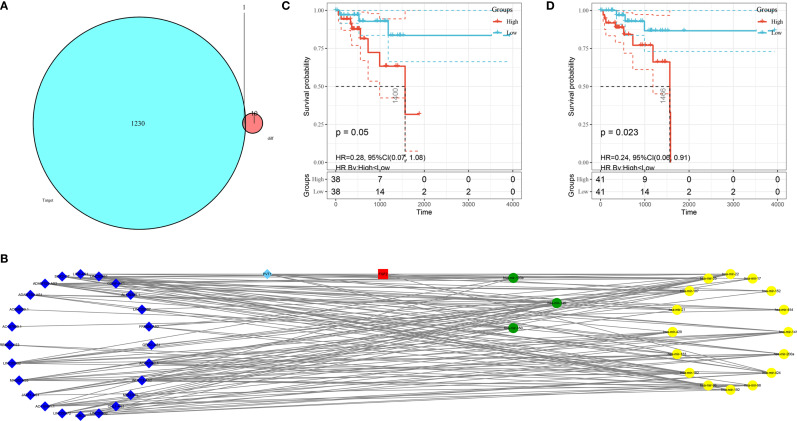
Construction of the ceRNA network and K–M plots of survival. **(A)** Venn diagram of the identified candidate mRNA. **(B)** ceRNA network based on the hub immune-related DEGs. The light blue diamonds show upregulated DElncRNAs; the dark blue diamonds show downregulated DElncRNAs; the yellow circles show upregulated DEmiRNAs; the green circles show downregulated DEmiRNAs; and the red rounded squares show mRNAs. **(C)** K–M plot of hsa-mir-107. The red line represents high hsa-mir-107 expression group, and the blue line represents the low hsa-mir-107 expression group. **(D)** K–M plot of WDFY3-AS2. The red line represents high expression of WDFY3-AS2, and the blue line represents low expression of WDFY3-AS2. K–M, Kaplan-Meier.

The mRNA, miRNA, and lncRNA characteristics are shown in **Table 2**. There were 24 downregulated DElncRNAs, 1 upregulated DElncRNA, 3 downregulated DEmiRNAs, 16 upregulated DEmiRNAs, and 1 candidate target mRNA (FGF2) in the network (**Figure 6A**). Based on the interactions between DEmiRNAs–DElncRNAs and DEmiRNAs–DEmRNAs, we built a DEmiRNA–DElncRNA–DEmRNA network (**Figure 6B**). Two biomarkers were associated with survival times: the miRNA hsa-mir-107 (P = 0.05, HR = 0.29, 95% CI: 0.07–1.08) and the lncRNA WDFY3-AS2 (P = 0.023, HR = 0.24, 95% CI: 0.06–0.91) (**Figures 6C, D** **)**. The biomarkers hsa-mir-107 and WDFY3-AS2 were not closely related to clinical information (**Supplementary Figures S5**, **S6**). There was no significant difference in the expression of has-miR-107 and WDFY3-AS2 in RC tumor and control tissues (**Supplementary Figure S7**).

**Table 2:** Basic information on the ceRNA network.

Dataset	No. of Normal tissues	No. of Tumor tissues	Platform ID	No. of Row Perl Platforms
mRNA	2	84	RNAseq	19,600
lncRNA	2	84	RNAseq	14,083
miRNA	1	78	RNAseq	1,881

miRNA, microRNA; lncRNA, long non-coding RNA.

### Development and Validation of the Prognostic Model

**Figure 7:**
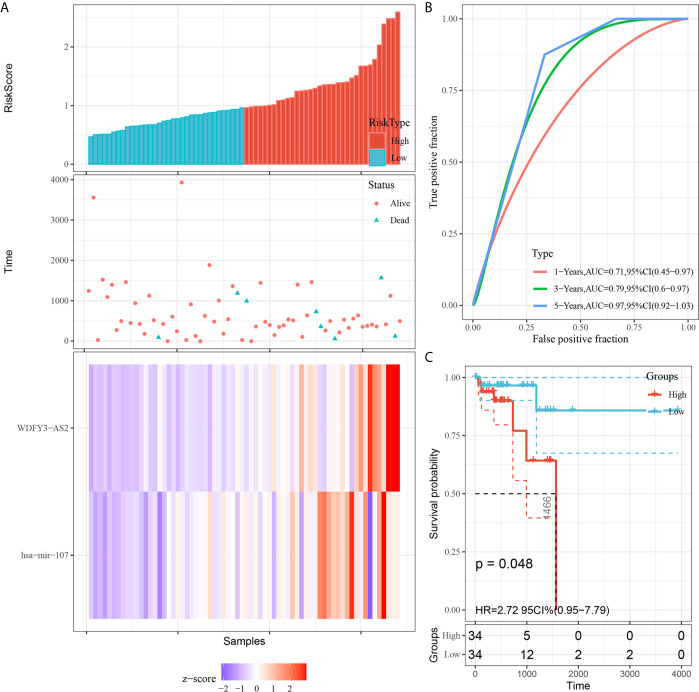
Construction of the prognostic model based on survival-related biomarkers. **(A)** Risk score distribution in RC patients. Red dots and lines represent the high-risk group of the model, and blue dots and lines represent the low-risk group of the model. **(B)** ROC curves of 1-, 3- and 5-year survival rates of RC patients. The red line represnts the 1-year survival rate of RC patients, the green line represents the 3-year survival rate, and the blue line represents the 5-year survival rate. **(C)** K–M plot of the risk score survival value.

**Figure 8:**
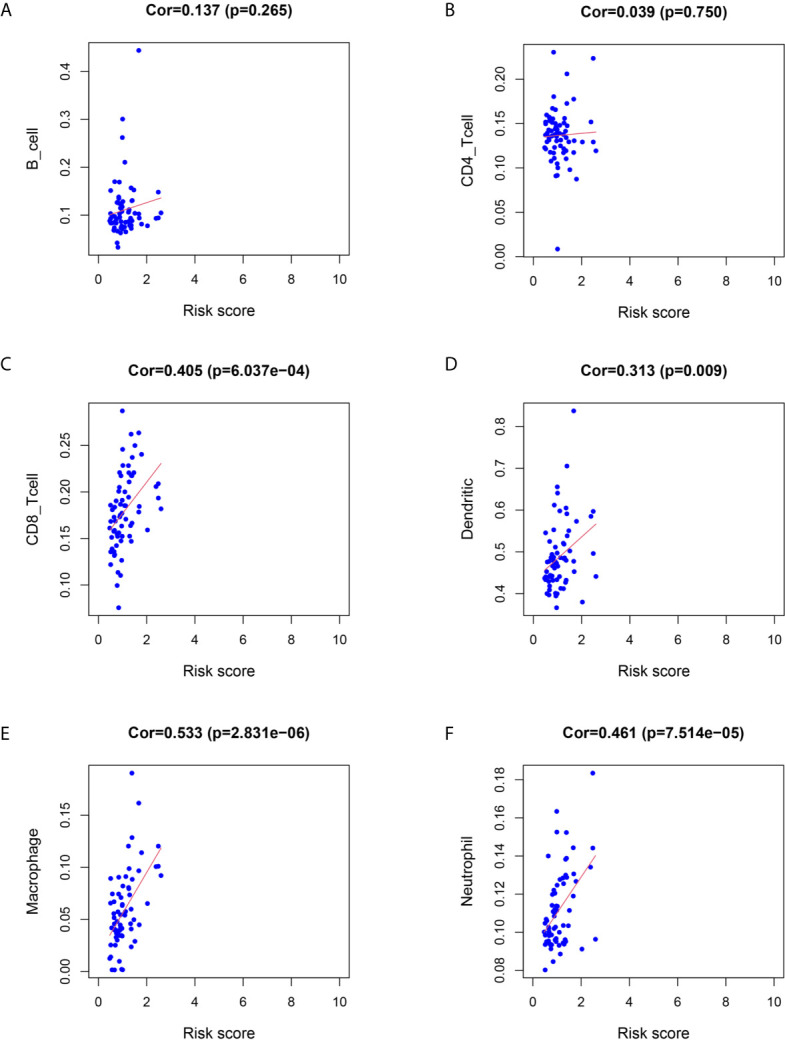
Relationship between immune cell infiltration and the risk score of the prognostic model. **(A–F)** was shown the correlationship between B cell, CD4 T cell, CD8 T cell, Dendritic cell, Macrophage, Neutrophil and risk score of prognostic model.

We utilized Cox proportional hazards regression analysis to develop the prognostic risk score model (**Figure 7**). Based on the risk score, we divided RC patients into low-risk and high-risk groups. The risk score distribution was analyzed and is shown in **Figure 7A**. The AUCs of 1-year survival, 3-year survival and 5-year survival were 0.71, 0.79, and 0.97, respectively (**Figure 7B**). We utilized K–M curves to show the relationship of the risk score with overall survival (OS). As shown in **Figure 7C**, the risk score had a strong relationship with OS, and the high-risk group had a poor prognostic value (P = 0.048). The risk score of the prognostic model had minimal relationship with B cells (cor = 0.137, P = 0.265) or CD4 T cells (cor = 0.039, P = 0.75). The prognostic model was significantly related to CD8-T cells (cor = 0.405, P = 6.037E-04), dendritic cells (cor = 0.313, P = 0.009), macrophages (cor = 0.533, P = 2.831E-06), and neutrophils (cor = 0.461, P = 7.514E-05) (**Figure 8**).

### Development and Validation of the Response-Related Prediction Nomogram

**Figure 9:**
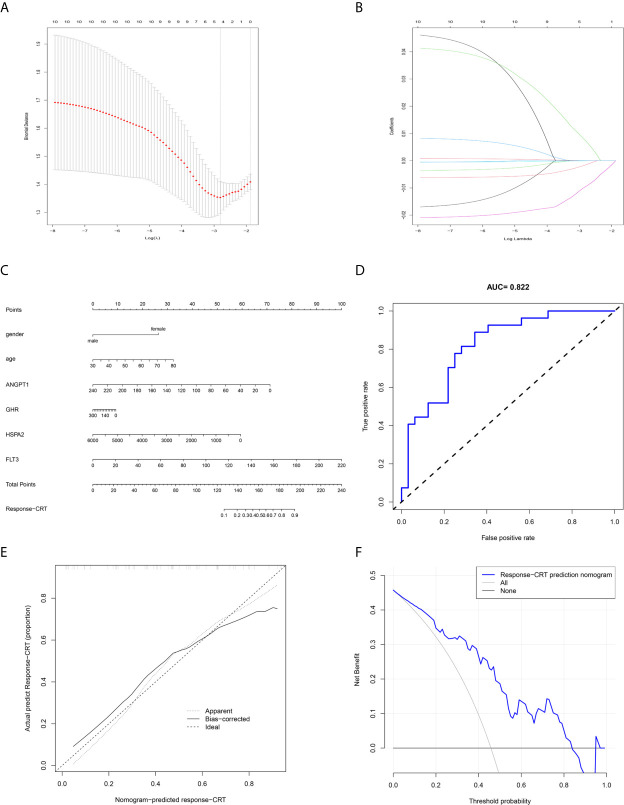
Prediction nomogram. **(A, B)** LASSO regression analysis of hub immune-related DEGs. **(C)** Prediction nomogram for the response to CRT in LARC patients. **(D)** ROC curve of the nomogram. **(E)** Calibration curve of the nomogram. The x-axis represents the predicted response-CRT risk. The y-axis represents the actual response to CRT. The solid line represents the performance of the nomogram, a closer fit to diagonal dotted line represents a better prediction. **(F)** DCA curve of the nomogram. The y-axis measures the net benefit. The blue line represents response-CRT nomogram. The gray line represents the assumption that all patients are responsive to CRT. The black line represents the assumption that no patients are responsive to CRT.

The response-related prediction nomogram (**Figure 9C**), which was built based on the response of LARC patients to CRT and LASSO regression analysis of the 10 hub immune response-related DEGs is shown in **Figures 9A, B**. Four genes were screened: ANGPT1, GHR, HSPA2, and FLT3. The C-index of the nomogram was 0.822 (SD = 0.107), the AUC of the ROC curve was 0.822 (**Figure 9D**), and the calibration and DCA plots (**Figures 9E, F**) showed good predictive value for the response of LARC patients to CRT. The verification C-index was 0.747, which suggested that the nomogram offers good discrimination.

## Discussion

CRC is a commonly diagnosed cancer and has high mortality (30). RC accounts for one-third of CRC cases worldwide (33). In recent years, CRT and surgery have been the standard treatments for LARC patients (34). Different responses to CRT are very important for LARC patients because of the close relationship between treatment and the survival rate (35). Therefore, it is necessary to develop an effective model to predict the therapeutic effect of CRT. Previously, Nie Ke et al. built a high predictive model based on multiparametric MRI (36), and some prognostic biomarkers were found to be linked with the response to CRT (37). IRGs as factors linked with tumor regression and clinical outcome (38), also play important roles in the response to CRT (39). In our study, we first explore the TME of RC. In our results, we found that CD4^+^ T cells and macrophages were enriched in RC tissue, and our gene microarray confirmed this result, which provided our perspective that immune infiltration may play an important role in RC. We continued to explore the IRDEGs based on the DEGs between normal and tumor tissue.

We identified 65 principal IRDEGs related to the response to CRT for LARC by PCA, such as IL6R (40) and CCL23 (41). Functional enrichment showed that IRDEGs were related to tumor regression. To further explore the biomarkers of CRT response, we identified the intersection between the biomarkers screened by PCA and RRA. Ten hub IRDEGs were identified and had close relationships with classic signaling pathways, such as the Ras signaling pathway, MAPK signaling pathway, and PI3K–Akt signaling pathway. To explore the potential biological value of these genes in LARC patients, we performed GO enrichment analyses to explore the functions, and most of the GO and KEGG terms were associated with the immune response and development of RC.

To explore the mechanisms of hub IRDEGs in response to CRT for LARC, we constructed a ceRNA network (42). miRNAs and lncRNAs are molecules that play important roles in regulating gene expression during normal or pathological cellular processes at the posttranscriptional level (43–45). In our study, we identified the DEmiRNAs and DElncRNAs between normal and tumor tissues and explored the candidate DEmRNAs based on the IRDEGs. In the ceRNA network, we found that miRNA hsa-mir-107 and lncRNA WDFY3-AS2 are associated with survival time and have a close relationship with the development and clinical outcome of cancer: hsa-mir-107 is a biomarker that is linked with the development of human cancer (46), and WDFY3-AS2 is linked with the tumor regression of ovarian cancer (47). There was no statistically significant difference in the expression of the two biomarkers in tumor and control tissues, which may indicate that the biological values of the two biomarkers are more inclined to prognosis. Meanwhile, the small numbers of specimens may also be the reason that affects the experimental results. Based on the two prognostic genes, we utilized Cox proportional hazards regression analysis to develop a prognostic model, and the model could predict the survival time very effectively. To analyze the relationship between tumor immune filtration and the prognostic model, we utilized six IRGs with reported links with tumors. To improve the nomogram prediction accuracy for LARC, we further screened 10 hub IRDEGs through LASSO regression, built a response-related prediction nomogram in the GSE68204 dataset, and validated the nomogram. All of the results showed that the nomogram offers good prediction value.

Our study also faces some limitations. First, a prognostic model nomogram was built based on the immune-related genes associated with the response of LARC patients to CRT, and the clinical information of LARC patients was not explored. In the future, we will add valuable clinical information to make the prognostic model and nomogram more comprehensive. Second, in the study, we explored the differential expression of the two prognostic biomarkers because the patients had experienced surgery and did not have enough time for follow-up studies. In the future, we will accumulate more clinical specimens and perform follow-up studies. We will further explore the mechanism of the response of LARC patients to CRT *in vivo* and validate the prognostic model in a large population.

In summary, by exploring the GEO and TCGA databases, which are publicly available, we identified immune-related genes, and based on the genes, we constructed a response-related prediction model and ceRNA network. We also found that the miRNA hsa-mir-107 and the lncRNA WDFY3-AS2 are associated with survival time and can be used as prognostic markers or treatment targets for LARC patients in the future. Based on these two genes, we built a prognostic risk score model. Our results PROVIDED new insights to predict the response to CRT for LARC.

## Data Availability Statement

The original contributions presented in the study are included in the article/**Supplementary Material**. Further inquiries can be directed to the corresponding author.

## Ethics Statement

The studies involving human participants were reviewed and approved by National Cancer Center/Cancer Hospital, Chinese Academy of Medical Sciences and Peking Union Medical College National GCP Center for Anticancer Drugs, The Independent Ethics Committee. The patients/participants provided their written informed consent to participate in this study.

## Author Contributions

F-ZW designed the research. Z-JW, S-WM, and T-XX organized the data. J-NC, H-YS, J-L, and F-QZ analyzed and visualized the data. F-ZW drafted the article. QL revised the paper. All authors contributed to the article and approved the submitted version.

## Funding

Key Project of National Key R & D Plan “Research on Prevention and Control of Major Chronic Non-Communicable Diseases” (No. 2019YFC1315705), China Cancer Foundation Beijing Hope Marathon Special Fund (No. LC2017L07), Medical and Health Science and Technology Innovation Project of the Chinese Academy of Medical Sciences (No. 2017-12M-1-006).

## Conflict of Interest

The authors declare that the research was conducted in the absence of any commercial or financial relationships that could be construed as a potential conflict of interest.

## Abbreviations

RC, rectal cancer; LARC, locally advanced rectal cancer; TCGA, The Cancer Genome Atlas; GEO, Gene Expression Omnibus; DEGs, differentially expressed genes; IRGs, immune-related genes; IRDEGs, immune-related differentially expressed genes; GO, gene ontology; KEGG, Kyoto Encyclopedia of Genes and Genomes; PCA, principal component analysis; RRA, robust rank aggregation; TNM, tumor node metastasis; LASSO, least absolute shrinkage and selection operator; DCA, decision curve analysis; ROC, receiver operating characteristic.
