# Supplementary material for: Development and Validation of a Nomogram and a Comprehensive Prognostic Analysis of an LncRNA-Associated Competitive Endogenous RNA Network Based on Immune-Related Genes for Locally Advanced Rectal Cancer With Neoadjuvant Therapy
Source: Front Oncol. 2021 Jul 19;11:697948. doi: 10.3389/fonc.2021.697948 (PMC8327778; doi:10.3389/fonc.2021.697948)
Supplement: Supplementary file 1 [file DataSheet_1.docx]

**Supplemental Materials and Methods**

**Immunofluorescence double staining:**

1. Deparaffinize and rehydrate: incubate sections in xylene(Sinopharm Chemical Reagent Co. Ltd)Ⅰfor 15min - xyleneⅡfor 15min - pure ethanolⅠfor15 min - pure ethanol(Sinopharm Chemical Reagent Co. Ltd)Ⅱfor 15 min - 85% ethanol for 5 min - 75% ethanol for 5 min - Wash in distilled water.
2. Antigen retrieval: immerse the slides in EDTA antigen retrieval buffer (pH 8.0) (G1203,Servicebio) and maintain at a sub-boiling temperature for 8 min - standing for 7 min - sub-boiling temperature for 7 min. Let air cooling. Wash three times with PBS (pH 7.4) (Servicebio,G0002) in a Rocker device, 5 min each.
3. Circle: After the section is slightly shaken dry, draw a circle around the tissue with a histochemical pen (to prevent the antibody from flowing away).
4. Block with serum: add BSA in circle and incubate for 30 min.
5. Add CD4^+^ antibody(first primary antibody): throw away the blocking solution slightly. Incubate slides with the CD4 Rabbit mAb(48274S, cst) (diluted with PBS appropriately:1:500) overnight at 4 ℃ in a wet box.
6. Corresponding Goat polyclonal Secondary Antibody to Rabbit IgG (First corresponding secondary antibody, Servicebio, GB25303) marked with HRP(1:500): wash slides three times with PBS (pH 7.4) in a Rocker device, 5 min each. Then throw away liquid slightly. Cover objective tissue with secondary antibody, incubate at room temperature for 50 min.
7. Add CY3 solution: wash slides with PBS (pH 7.4) in a Rocker device, three times, 5 min each. Incubate with CY3(Servicebio) for 10 min in dark condition. Then wash slides three times with TBST in a Rocker device, 5 min each.
8. Microwave treatment: immerse the slides in EDTA antigen retrieval buffer (pH 8.0) and maintain at a sub-boiling temperature for 8 min - standing for 8 min - sub-boiling temperature for 7 min.
9. Add CD68 XP Rabbit mAb(second primary antibody): Incubate slides with CD68 XP Rabbit mAb(diluted with PBS appropriately:1:200) overnight at 4 ℃, placed in a wet box.
10. Corresponding Goat polyclonal Secondary Antibody to Rabbit IgG with 488 (Second corresponding secondary antibody): wash slides three times with PBS (1:400, pH 7.4) in a Rocker device, 5 min each. Then throw away liquid slightly. Cover objective tissue with secondary antibody (appropriately respond to second primary antibody in species), incubate at room temperature for 50 min in dark condition.
11. Spontaneouss fluorescence quenching: eliminate obvious liquid, add spontaneouss fluorescence quenching reagent in circles for 5 min, then wash slides under flowing water for 10 min.
12. DAPI counterstain in nucleus: add DAPI solution in circles at room temperature for 10 min in dark condition.
13. Mount: wash three times with PBS (pH 7.4) in a Rocker device, 5 min each. Throw away liquid slightly, then coverslip with anti-fade mounting medium.
14. Microscopy detection and collect images by Fluorescent Microscopy. DAPI glows blue by UV excitation wavelength 330-380 nm and emission wavelength 420 nm; FITC glows green by excitation wavelength 465-495 nm and emission wavelength 515-555 nm;  CY3 glows red by excitation wavelength 510-560 nm and emission wavelength 590 nm.

**Real-time quantitative polymerase chain reaction (qPCR):**

**The sequences of the primers were as follows:**

WDFY3-AS2: sense primer 5’-CGCAAAGGCTACTAGACGCA-3’

antisense primer 5’-CATTGGCTTGCCCAGTTTCT-3’

GAPDH: sense primer 5’-GGAAGCTTGTCATCAATGGAAATC-3’

antisense primer 5’-TGATGACCCTTTTGGCTCCC-3’

Hsa-mir-017: sense primer 5’-ACACTCCAGCTGGGAGCAGCATTGTACAGGG-3’

antisense primer 5’-CTCAACTGGTGTCGTGGAGTCGGCAATTCAGTTGAGTGATAGCC-3’

U6: sense primer 5’-CTCGCTTCGGCAGCACA-3’

antisense primer 5’-AACGCTTCACGAATTTGCGT-3’

Universal primer A: 5’-TGGTGTCGTGGAGTCG-3’ (used for amplification with miRNA sense primer).

**qPCR procedure:**

Isolate RNA

1. Take homogenate tube, add 1ml RNA Extraction (Servicebio, G3013) and put on ice to precool.
2. Take 100mg of tissue into the homogenate tube.
3. The tissues were ground with High-speed-microtherm Homogenizer (Servicebio, KZ-III-F) until no visible tissues mass.
4. Centrifuge (DragonLab, D3024R) the samples at 12000rpm for 10 min, transfer the supernatant to a new tube.
5. Add 0.25ml of chloroform (Chemical Reagent Co.Ltd, 10006818) and cap sample tubes securely. Vortex samples vigorously for 15 seconds adn incubate samples at room temprerature for 3 min.
6. Centrifuge the samples at 12000rpm for 10 min at 4 ℃.
7. Transfer 400μl supernatant to new tubes, add 0.8 volume of iso-Propyl alcohol (Chemical Reagent Co. Ltd, 80109218) and mix upside down.
8. Incubate samples at -20 ℃ for 15 min.
9. Centrifuge the samples at 12000rpm for 10 min at at 4 ℃. The white sediment at the bottom of the tubes is RNA.
10. Remove the liquid completely and wash the RNA sediment with 1.5 ml 75% ethanol (Chemical Reagent Co.Ltd, 10009218).
11. Centrifuge the samples at 12000rpm for 10 min at at 5 ℃.
12. Remove the liquid completely, place the centrifuge tube on the Clean bench and blow for 3 min.
13. Dissolve RNA with 15μl DEPC-treated water.
14. Incubate samples at 55 ℃ for 5 min.
15. Use Ultra-micro spectrophotometer (Thermo, NanoDrop 2000) to detect RNA concentration and purity: After blank zeroing of the instrument, take 2.5μl RNA solution to be tested on the detection base, put down the sample arm, and used the software on the computer to test the absorption value. The excessively high concentration of RNA was diluted to a proper proportion, so that the final concentration was 100-500 ng/μl.

First Strand cDNA Synthesis

1. Preparation of reverse transcription reaction system (20μl)

| **Component** | **Volume** |
| --- | --- |
| 5 x Reaction Buffer | 4 μL |
| Gene-specific primer(0.5μM) | 2 μL |
| Servicebio®RT Enzyme Mix | 1 μL |
| Total RNA * | 10 μL |
| RNase free water | Add to 20 μL |

1. Mix gently and centrifuge briefly.
2. Reverse transcription

| **Temperature** | **Time** |
| --- | --- |
| 25℃ | 5 min |
| 42℃ | 30 min |
| 85℃ | 5 sec |

Preparation of PCR Master Mix

1. For each 15 μl reaction, prepare the following reation mix:
2. 2X qPCR Mix (2×SYBR Green qPCR Master Mix (None ROX), Servicebio,G3320): 7.5 μl
3. Primer (2.5μM): 1.5 μl
4. cDNA template: 2.0 μl
5. ddH_2_O: 4.0 μl
6. PCR amplification
7. Pre-denaturation: 95℃, 10 min.
8. Cycle (40 times): 95℃, 15s 60℃, 60s
9. Melt Curve: 60℃ 95℃, fluorescence signal was collected once for each 0.5℃ temperature rise.

Results of processing

ΔΔCT method:

A = CT (target gene, sample) - CT(internal standard gene, sample)

B = CT (target gene, control) - CT(internal standard gene, control)

K = A - B

RNA Expression = 2^-K^
