# Supplementary figures and images for: Development and Validation of a Nomogram and a Comprehensive Prognostic Analysis of an LncRNA-Associated Competitive Endogenous RNA Network Based on Immune-Related Genes for Locally Advanced Rectal Cancer With Neoadjuvant Therapy
Source: Front Oncol. 2021 Jul 19;11:697948. doi: 10.3389/fonc.2021.697948 (PMC8327778; doi:10.3389/fonc.2021.697948)

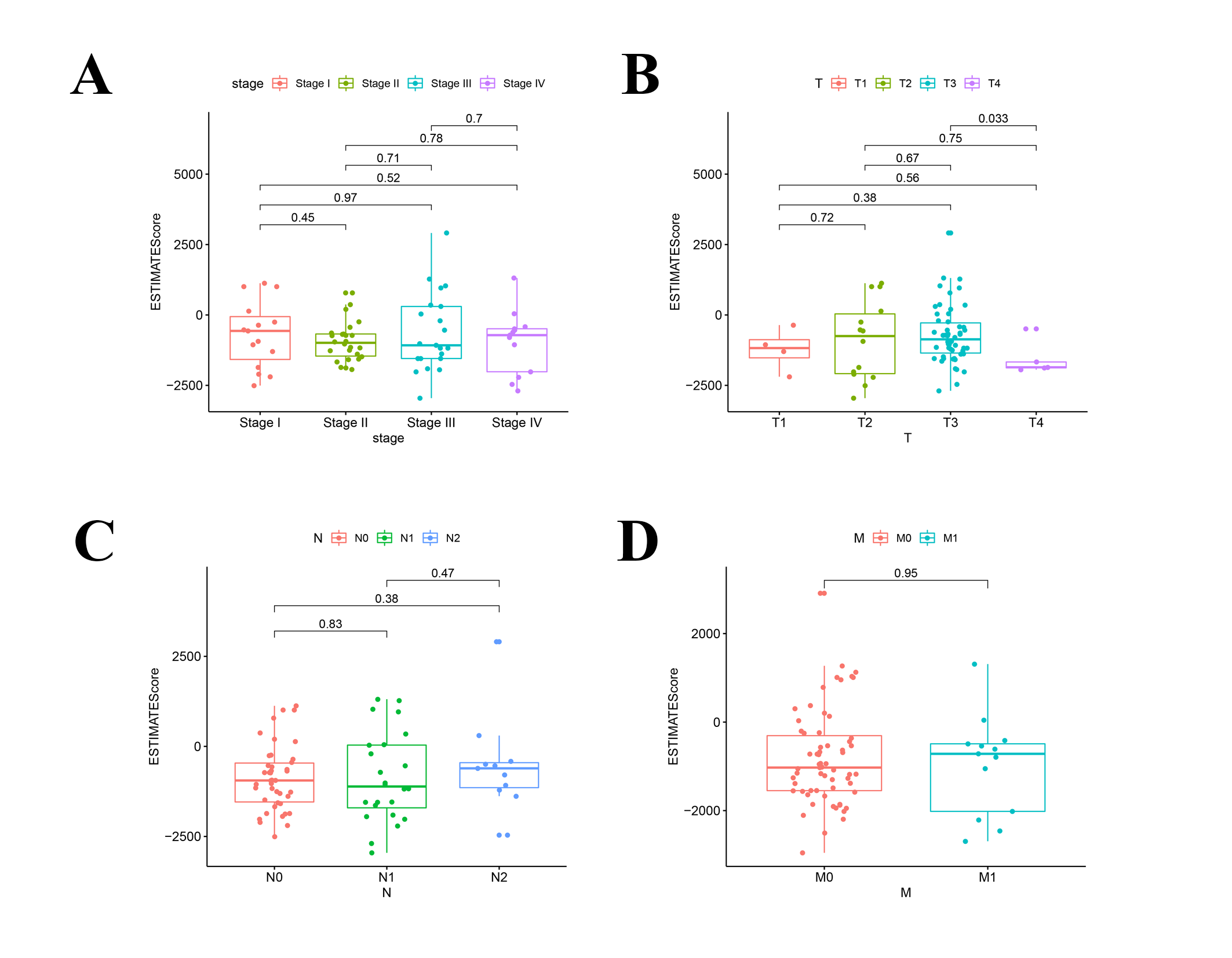

Supplement: Supplementary file 2 [file Image_1.tif]

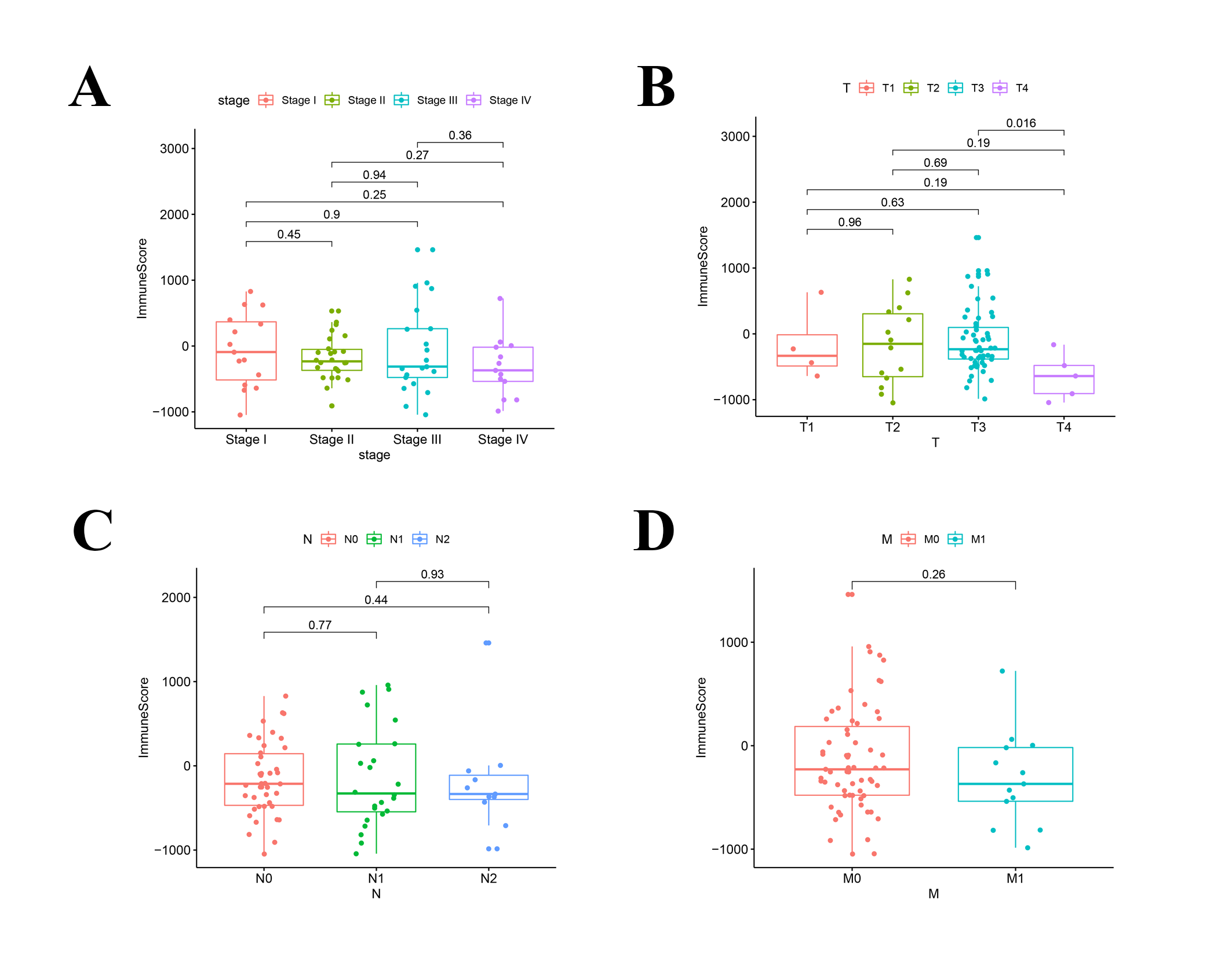

Supplement: Supplementary file 3 [file Image_2.tif]

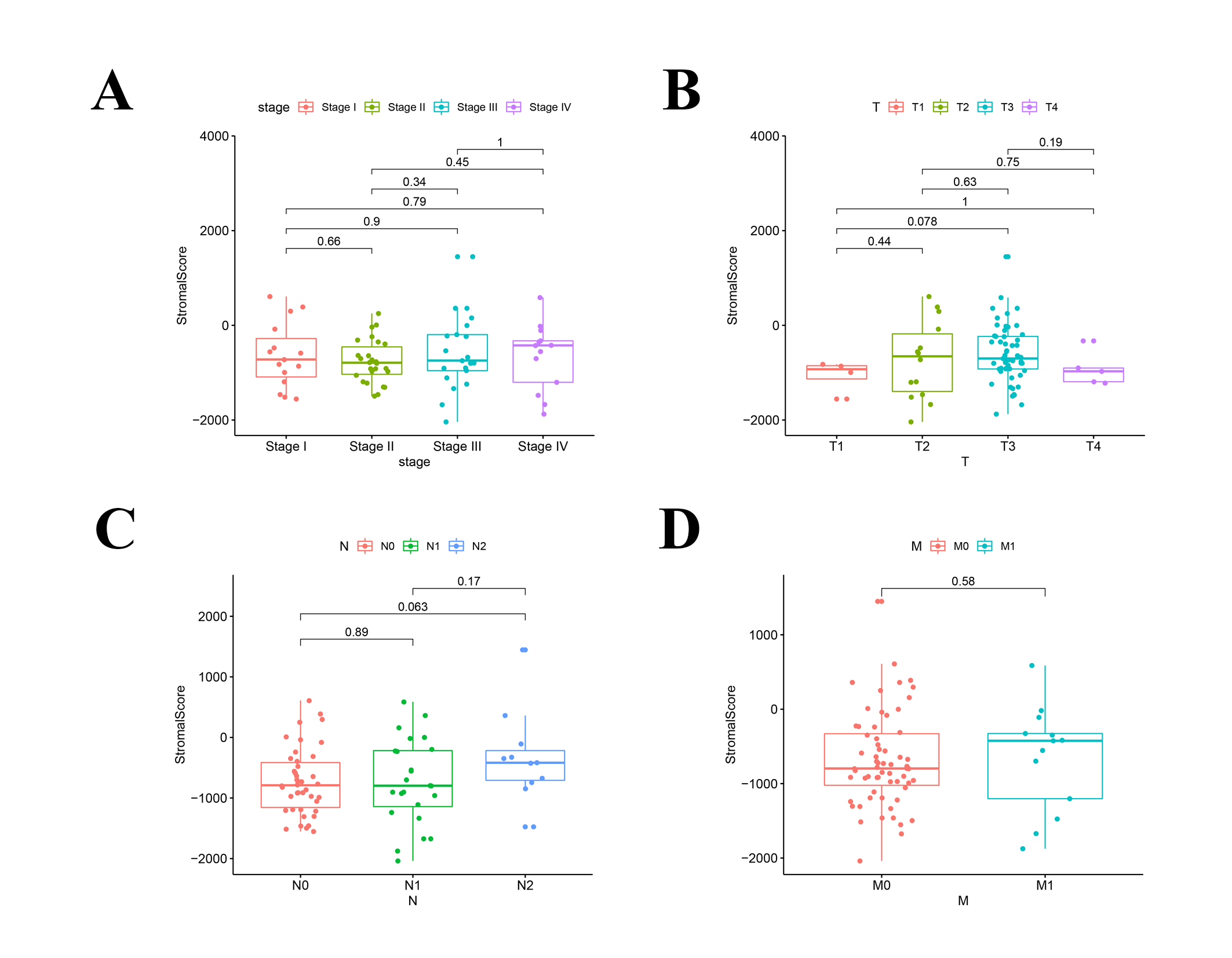

Supplement: Supplementary file 4 [file Image_3.tif]

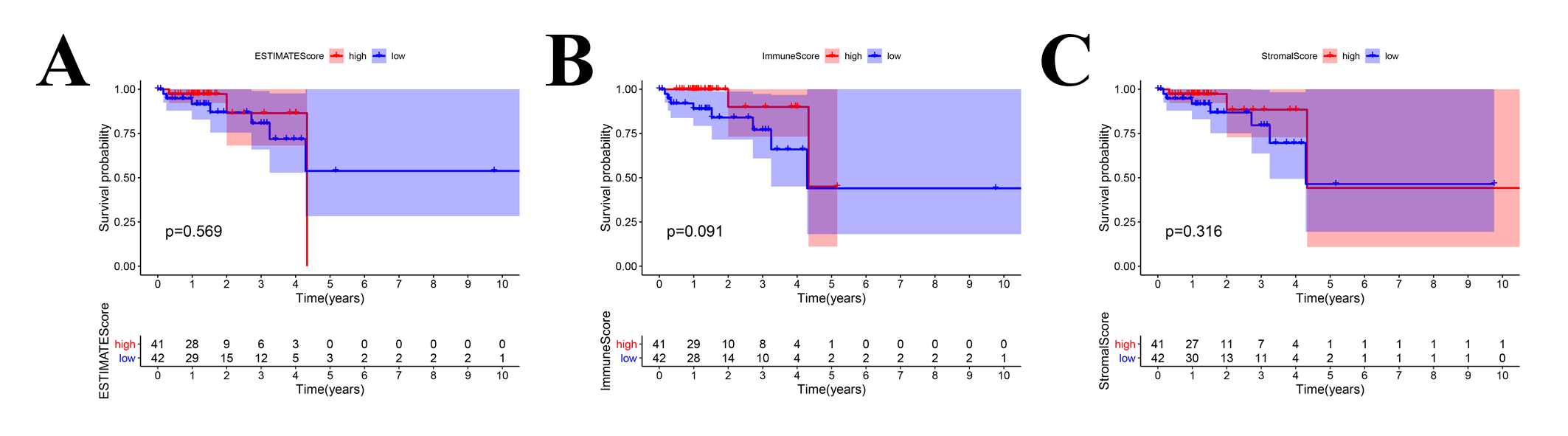

Supplement: Supplementary file 5 [file Image_4.tif]

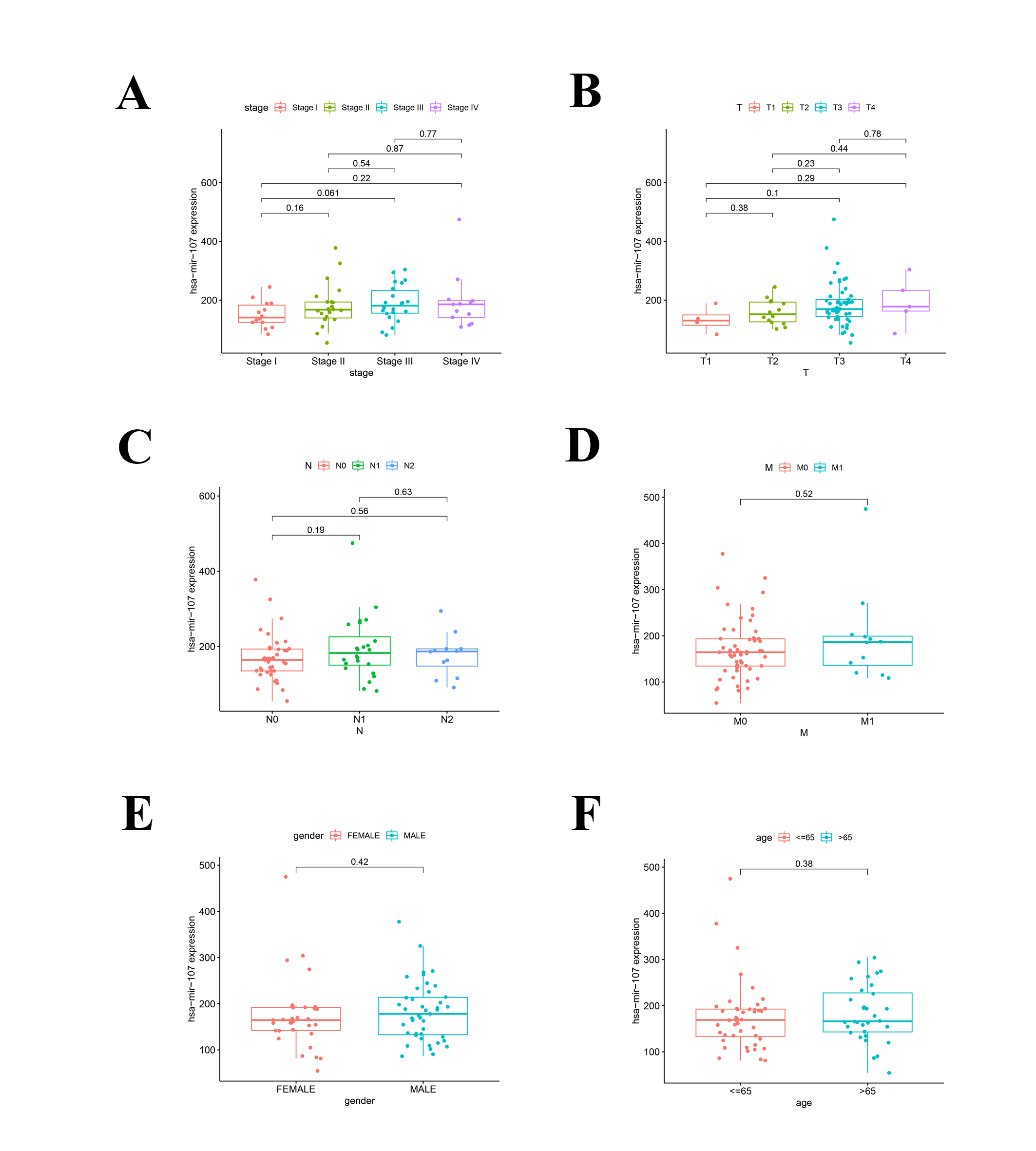

Supplement: Supplementary file 6 [file Image_5.tif]

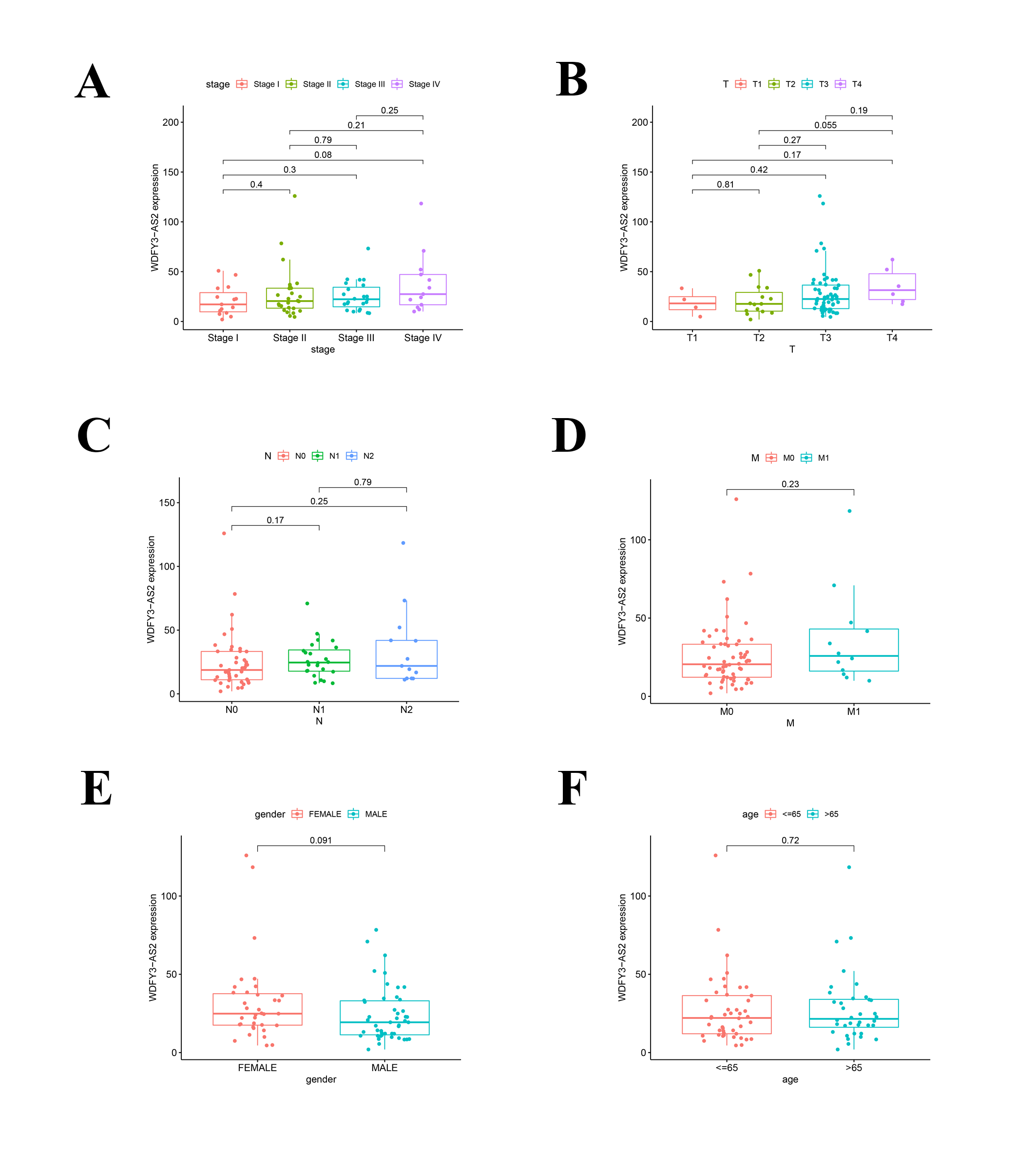

Supplement: Supplementary file 7 [file Image_6.tif]

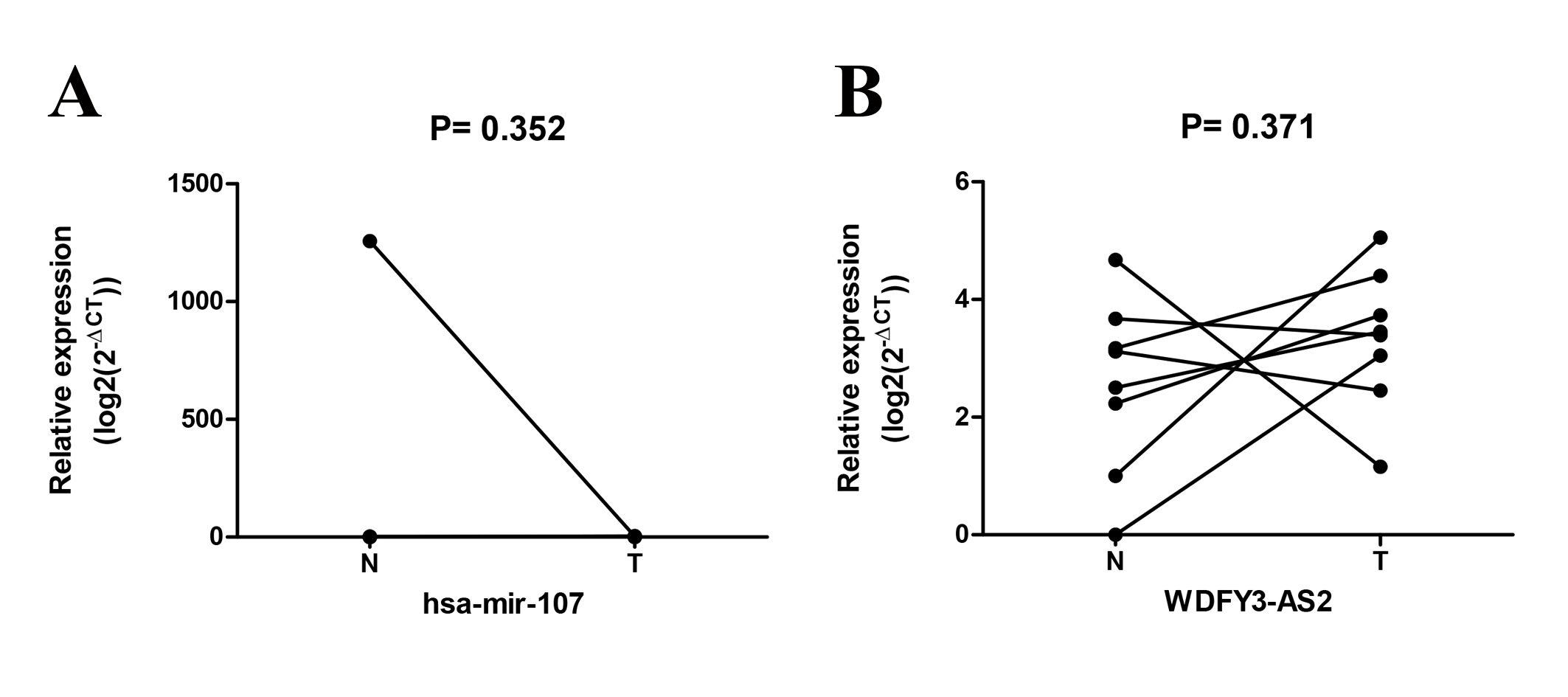

Supplement: Supplementary file 8 [file Image_7.tif]
